# Inhibition of endogenous SPARC enhances pancreatic cancer cell growth: modulation by FGFR1-III isoform expression

**DOI:** 10.1038/sj.bjc.6605440

**Published:** 2009-11-17

**Authors:** G Chen, X Tian, Z Liu, S Zhou, B Schmidt, D Henne-Bruns, M Bachem, M Kornmann

**Affiliations:** 1 Clinic of General, Visceral, and Transplantation Surgery, University of Ulm, Steinhoevelstrasse 9, Ulm 89075, Germany; 2 Department of Clinical Chemistry, University of Ulm, Steinhoevelstrasse 9, Ulm 89075, Germany

**Keywords:** osteonectin, SPARC, fibroblast growth factor receptor, pancreatic cancer, pancreatic stellate cells

## Abstract

**Background:**

Secreted protein acidic and rich in cysteine (SPARC) is a multi-faceted protein-modulating cell–cell and cell–matrix interactions. In cancer, SPARC can be not only associated with a highly aggressive phenotype, but also acts as a tumour suppressor. The aim of this study was to characterise the function of SPARC and its modulation by fibroblast growth factor receptor (FGFR) 1 isoforms in pancreatic ductal adenocarcinoma (PDAC).

**Methods and results:**

Exogenous SPARC inhibited growth, movement, and migration. ShRNA inhibition of endogenous SPARC in ASPC-1 and PANC-1 cells resulted in increased anchorage-dependent and -independent growth, transwell migration, and xenograft growth as well as increased mitogenic efficacy of fibroblast growth factor (FGF) 1 and FGF2. Endogenous SPARC expression in PANC-1 cells was increased in FGFR1-IIIb over-expressing cells, but decreased in FGFR1-IIIc over-expressing cells. The up-regulation of endogenous SPARC was abrogated by the p38-mitogen-activated protein kinase inhibitor SB203580. SPARC was detectable in conditioned medium of pancreatic stellate cells (PSCs), but not PDAC cells. Conditioned medium of PDAC cells reduced endogenous SPARC expression of PSCs.

**Conclusion:**

Endogenous SPARC inhibits the malignant phenotype of PDAC cells and may, therefore, act as a tumour suppressor in PDAC. Endogenous SPARC expression can be modulated by FGFR1-III isoform expression. In addition, PDAC cells may inhibit endogenous SPARC expression in surrounding PSCs by paracrine actions.

Secreted protein acidic and rich in cysteine (SPARC) or osteonectin is a 32–33 kDa calcium-binding glycoprotein shown to associate with the cell membrane and membrane receptors (Yan and Sage, 1999). It belongs to a family of matricelluar proteins and is divided into three modules that exert various functions (Bradshaw and Sage, 2001). It functions not only to modulate cell–cell and cell–matrix interactions, but also has de-adhesive and growth inhibitory properties in non-transformed cells (Tai and Tang, 2008). In cancer, SPARC may exert divergent actions reflecting the complexity of this protein (Clark and Sage, 2008; Podhajcer *et al*, 2008). Thus, in certain types of cancers, such as melanomas and gliomas, SPARC is associated with a highly aggressive tumour phenotype, whereas in others, mainly ovarian, neuroblastomas, and colorectal cancers, SPARC may function as a tumour suppressor (Tai and Tang, 2008). These functions are thought to be exerted in part by its ability to counteract effects of several growth factor families including fibroblast growth factors (FGFs) (Yan and Sage, 1999; Francki *et al*, 2003; Motamed *et al*, 2003).

Modulation of FGF actions by SPARC is mediated by FGF receptor (FGFR) 1, because FGFR1 was reported to be indispensable for SPARC-induced inhibition of FGF signalling (Motamed *et al*, 2003). The presence of several FGFR1 isoforms generated by alternative mRNA splicing makes the analysis of FGFR1 and SPARC interactions difficult. FGF actions strongly depend on the presence of specific FGFR isoforms and can be modulated by changes in isoform expression (Ornitz *et al*, 1996; Kornmann *et al*, 2001; Liu *et al*, 2007a). Alternative splicing of the second half of Ig-like domain III generates particular important isoforms, because this region determines ligand-binding specificity (Ornitz *et al*, 1996; Plotnikov *et al*, 2000). As a consequence, over-expression of FGFR1-IIIc in pancreatic cancer promoted tumourigenesis, whereas over-expression of FGFR1-IIIb inhibited the malignant phenotype (Kornmann *et al*, 2002; Liu *et al*, 2007b). In addition, several FGFs, signalling through FGFR1-IIIc, are highly over-expressed in human pancreatic ductal adenocarcinoma (PDAC) (Yamanaka *et al*, 1993). Several recent studies reported increased SPARC levels in human PDAC (Sato *et al*, 2003; Guweidhi *et al*, 2005) associated with poor prognosis (Infante *et al*, 2007). SPARC expression was absent in most of the cancer cells, but instead present at high levels in the peritumoural tissue harbouring fibroblasts and pancreatic stellate cells (PSCs) (Guweidhi *et al*, 2005; Infante *et al*, 2007).

Despite these recent efforts, the functions of SPARC and its associations with FGFR1-III isoforms in PDAC remain unclear. Therefore, the aim of this study was to elucidate SPARC functions and endogenous SPARC regulation depending on FGFR1-III isoforms expression in PDAC cells and its interaction with PSC cells.

## Materials and methods

### Cell culture

ASPC-1, BXPC-3, CAPAN-1, MIA PaCa-2, and PANC-1 human pancreatic cancer cells were purchased from American Type Culture Collection (ATCC, Manassas, VA, USA), COLO357 and T3M4 human pancreatic cancer cells were a gift from M Korc (Dartmouth Medical School, Hanover, NH, USA). Human PSC were isolated and characterised as described earlier (Bachem *et al*, 2005). PANC-1, Mia PaCa-2, and COLO357 cells were grown in DME medium and ASPC, BXPC-3, CAPAN-1, and T3M4 cells in RPMI 1640 medium supplemented with 10% foetal bovine serum (FCS), penicillin G (100 units ml^–1^), and streptomycin (100 *μ*g ml^–1^) termed as complete medium. PSCs were grown in complete DME/F12 (1 : 1) medium supplemented with Amphotericin B (250 *μ*g ml^–1^). The respective media supplemented with 0.1% BSA, 5 mg l^–1^ transferrin, 5 *μ*g l^–1^ selenium, penicillin G (100 units ml^–1^), and streptomycin (100 *μ*g ml^–1^) were used as serum-free media. PANC-1 clones PF4 and PF40 (over-expressing FGFR1-IIIb), and PFc18 and PFc51 (over-expressing FGFR1-IIIc) were grown in complete DME medium with G418 (1.2 mg ml^–1^). SPARC shRNA-transfected cells were grown in complete RPMI and DME medium with G418 (0.8 and 1.2 mg ml^–1^, respectively) for ASPC-1 and PANC-1. Cells were maintained in monolayer culture at 37°C in humidified air with 5% CO_2_.

### Establishment of cell clones over-expressing FGFR1-III variants

The establishment of the FGFR1-IIIb and -IIIc PANC-1 clones over-expressing the full-length cDNA of human FGFR1-IIIb or -IIIc expressed in a modified pSVK3 vector under the control of the simian virus 40 early promotor in a stable manner was described earlier (Liu *et al*, 2007b; Chen *et al*, 2008).

### Establishment of cell clones expressing SPARC shRNA

Validated SureSilencing human SPARC shRNA and control plasmids were from SuperArray Bioscience Corp. (Frederick, MD, USA). ASPC-1 and PANC-1 cells were transfected in a stable manner using lipofectamine (Invitrogen, Carlsbad, CA, USA) following the manufacturer's protocol. Transfected cells were selected with G418 (0.8 and 1.2 mg ml^–1^ for ASPC-1 and PANC-1, respectively) for 14 days before isolation of individual clones.

### Immunoblot analysis

Total cell lysates were prepared and followed by immunoblot analysis as described (Liu *et al*, 2007b). A rabbit polyclonal antibody (SPARC, sc-25574, from Santa Cruz) was used (1 : 200) to detect SPARC protein. Bound antibodies were visualised using enhanced chemiluminescence. To confirm equal loading, membranes were stripped for 30 min at 50°C in buffer containing 2% SDS, 62.5 mM Tris (PH 6.7), and 100 mM 2-mercaptoethanol and reprobed with an anti-*β*-actin antibody to show equal loading.

### RT–PCR

Total RNA (1–2 *μ*g) was reverse transcribed using a SuperScript pre-amplification kit (Invitrogen). Primers were based on the sequences reported on Genebank (NM 003118). SPARC sense sequence was 5′-GTGGGCAAAGGGAAGTAACA-3′ and SPARC anti-sense sequence 5′-GGGAGGGTGAAGAAAAGGAG-3′. The expected product size of SPARC cDNA was 514 bp. PCR amplification was performed in 25 *μ*l reaction volumes containing 0.2 *μ*M dNTPs, 20 pmol of each oligonucleotide primer, and 0.2 U Tag polymerase in PCR buffer. cDNA was amplified on a PCR thermal controller with an initial denaturation at 95°C for 5 min, followed by cycles of 95°C for 1 min, 65°C for 1 min, and 72°C for 1 min, 27 cycles, and a final extension step of 72°C for 10 min. The amount of starting cDNA was adjusted using *β*-actin intensity.

### Anchorage-dependent growth and functional assays

The effects of exogenous SPARC (from murine parietal yolk sac cells, S5174 Sigma, St Louis, MO, USA) on proliferation were determined by the 3-(4,5-dimethylthiazol-2-yl)-2,5-diphenyltetrazolium bromide (MTT) assay. The indicated amount of cells was seeded in 96-well plates and propagated for 24 h in complete medium before further analysis. To assess basal growth and the effect of SPARC alone, 5000 cells per well were incubated for 48 h in complete medium in the absence or presence of SPARC. To assess the effects of SPARC on FGF-induced proliferation, cells (10 000 per well) were propagated for 48 h in serum-free medium including the indicated factors. FGF1 (recombinant human FGF acidic, R&D Systems) and FGF2 (recombinant human FGF basic, R&D Systems) were added in the presence of heparin (1 *μ*g ml^–1^).

To analyse N-linked protein, glycosylation cells were incubated in the absence or presence of tunicamycin (5 *μ*g ml^–1^, T7765 Sigma) for 24 h as described (Liu *et al*, 2007a). To assess the effect of kinase, inhibitors cells were incubated in the absence or presence of the p38-mitogen-activated protein kinase (MAPK) inhibitor SB203580 (Calbiochem, Darmstadt, Germany) and the c-Jun N-terminal kinase (JNK) inhibitor SP600125 (Calbiochem) for 24 h (Liu *et al*, 2007a).

### Single cell movement

Cells (50 000 per well) were seeded onto fibronetin-coated (5 *μ*g ml^–1^ in PBS) six-well plates and grown for 20 h. Cells were then incubated in the presence or absence of SPARC (5 *μ*g ml^–1^) for 24 h. Cell movement during that period was monitored by an Olympus IX81 motorised inverted microscope taking pictures every 10 min (Liu *et al*, 2007b). The total distance of individual cells covered within 24 h was determined using the ImageJ 1.32 program (NIH, Bethesda, MD, USA).

### Cell migration assay

The ability of cells to migrate through filters was measured using a BioCoat Matrigel invasion chamber (BD Biosciences, San Jose, CA, USA). Cell culture inserts with an 8 *μ*m pore size PET membrane were used according to the protocol of the manufacturer. The bottom chamber included medium (0.75 ml) containing 10% FCS, whereas cells (1.0 × 10^5^ suspended in 0.5 ml of medium containing 1% FCS) were seeded into the upper chamber and incubated overnight at 37°C in a humidified atmosphere containing 5% CO_2_. Cells were then incubated in the absence or presence of exogenous SPARC (5 *μ*g ml^–1^) for another 24 h. Remaining cells on the upper surface were mechanically removed. Membranes were then washed, fixed, and stained by Diff-Quik (Medion Diagnostics, Düdingen, Switzerland). The number of cells that migrated to the lower surface of the filters was determined by counting stained cells under a light microscope.

### Anchorage-independent growth assay

Basal anchorage-independent cell growth was assessed by a double-layer soft-agar assay as described (Kornmann *et al*, 2002). Briefly, cells were suspended in complete medium containing 0.3% agar and seeded in triplicate in six-well plates onto a base layer of complete medium containing 0.5% agar. One mililiter of complete medium containing 0.3% agar was added every 5 days. After 14 days, 300 *μ*g MTT per well was added to stain vital colonies for 24 h before counting by microscopy.

### *In vivo* tumourigenicity assay

To assess the effect of expression of SPARC on xenograft formation, 10^6^ cells per site were injected s.c. into two sites of 4- to 6-week-old female athymic (nude) mice. Animals were monitored for tumour formation every 4 days. Tumour size was measured in three dimensions. Tumour volume was determined by the equation vol=*l* × *w* × *d* × 0.5, where *l* is the length, *w* is the width, and *d* is the diameter. Animals had to be killed 12 weeks after injection according to our animal protocol (#718) if neither tumour volume (>2 cm^3^) nor skin ulcerations prompted earlier termination.

### Immunohistochemistry of xenograft tumours

SPARC expression in control-transfected (*n*=10) and FGFR1-IIIb over-expressing (*n*=10) xenograft tumours (Liu *et al*, 2007b) was determined by immunohistochemical analysis. SPARC immunohistochemistry was performed using formalin-fixed and paraffin-embedded sections using a rabbit polyclonal antibody detecting human SPARC (1 : 2000, sc-25574 from Santa Cruz) in combination with a secondary biotynylated goat anti-rabbit antibody and a Vectastain Elite ABC kit (Vector Lab, Burlingame, CA, USA) according to the protocol of the manufacturer.

### Detection of SPARC in conditioned medium

Indicated cells were grown in complete medium to 70% confluency in 10 cm dishes. After washing twice with PBS, cells were incubated for 48 h in 10 ml of serum-free medium containing proteinase inhibitors as described (Kornmann *et al*, 1997). For immunoblot analysis, conditioned medium of five dishes was collected and incubated at 4°C overnight after adjusting the pH to 7.4 and adding 50 *μ*l slurry heparin sepharose (CL-6B, Pharmacia Biotech, Piscataway, NY, USA) to pull-down calcium-binding proteins (Kornmann *et al*, 1997). The beads were collected by centrifugation, washed three times with 0.45 M NaCl/20 mM Tris–HCl (pH 7.4), and resuspended in 2 × Laemmli buffer. Samples were boiled for 5 min and subjected to immunoblot analysis.

### Statistics

The results were expressed as mean expression levels (±s.d. or s.e.m.). Student's *t*-test or rank sum test was used for statistical analysis. A *P*-value <0.05 was taken as level of significance (two sided).

## Results

### Expression of SPARC and its effects on proliferation and migration in cultured PDAC cells

**Figure 1:**
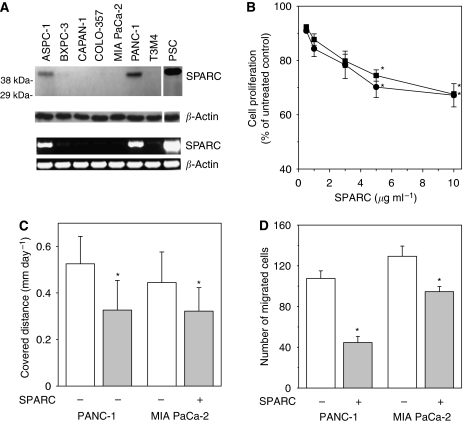
SPARC expression and its effects on proliferation, single cell movement, and migration. (**A**) SPARC protein (upper panel) and mRNA (lower panel) expression in cultured PDAC cells. Human PSC were used as a positive control. *β*-actin served as loading control. (**B**) Cell proliferation. COLO-357 (▪) and PANC-1 (•) cells were cultured in complete medium with increasing concentrations of SPARC. Cell proliferation was determined after 48 h by the MTT assay. The results are shown as mean growth (in %) of untreated control and are means (±s.e.) of quadruplicate determinations from three separate experiments. ^*^P<0.05. (**C**) Single cell movement. Movement of single cells was monitored by time-lapse microscopy taking pictures every 10 min for 24 h. The distance of individual cells (*n*=25) was evaluated using the ImageJ 1.32 and Simple Track programme. The results are shown as mean distances (±s.d.) in mm covered within 24 h in the absence (−) or presence (+) of SPARC (5 *μ*g ml^–1^). ^*^P<0.05. (**D**) Migration assay. The number of cells that moved to the lower side of the Boyden chamber was determined in the absence (−) or presence (+) of SPARC (5 *μ*g ml^–1^). The results are expressed as mean number (±s.d.) of migrated cells within 36 h of three separate experiments. ^*^P<0.05.

A clear signal of SPARC protein, migrating under reducing conditions at 43 kDa, and mRNA was found in ASPC-1 and PANC-1 cells (Figure 1A), whereas very week signals of SPARC mRNA were observed in the remaining five cell lines (Figure 1A, lower panel). In contrast, higher levels of SPARC were found in human PSCs (Figure 1A). Independent of endogenous SPARC expression, exogenous SPARC inhibited proliferation of PDAC cells. This effect was most pronounced in COLO-357 and PANC-1 cells (Figure 1B). Exogenous SPARC (5 *μ*g ml^–1^) also reduced proliferation of CAPAN-1 (−21±0.1% s.e.m.) and MIA PaCa-2 cells (−20±1.8% s.e.m.), but was without marked effect in ASPC-1 (−12±0.4% s.e.m.), BxPC-3 (−7.5±1.1% s.e.m.), and T3M4 cells (0.7±2.1% s.e.m.).

The effects of exogenous SPARC (5 *μ*g ml^–1^) on single cell movement and migration were investigated in PANC-1 and MIA PaCa-2 cells. SPARC reduced the distance covered within 24 h by 38% and 28% in PANC-1 and MIA PaCa-2 cells, respectively (Figure 1C). Cell migration was inhibited in the presence of SPARC by 58% and 26%, respectively (Figure 1D).

### Inhibition of endogenous SPARC expression

**Figure 2:**
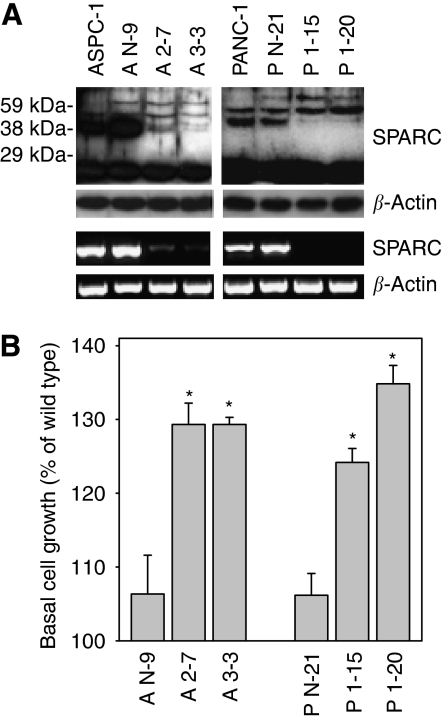
SPARC shRNA expression and its effect on basal cell growth. (**A**) SPARC protein (upper panel) and mRNA (lower panel) expression in wild-type ASPC-1 and PANC-1 cells, vector alone (control)-transfected clones (AN-9, PN-21), and SPARC shRNA-expressing clones (A2-7, A3-3, P1-15, P1-20), respectively. *β*-actin served as loading control. The long exposure autoradiographs for the SPARC immunoblot analysis revealed that still faint bands of SPARC are detectable in shRNA-expressing clones. (**B**) Basal cell proliferation. Basal growth was determined after 48 h in complete medium by the MTT assay. The results are shown as mean growth (in %) of the respective wild-type cell lines and are means (±s.e.) of quadruplicate determinations from six separate experiments. ^*^P<0.05 compared with wild-type and control-transfected cells.

To determine the importance of endogenous SPARC expression for the malignant phenotype of cultured PDAC cells, ASPC-1 and PANC-1 cell clones were established expressing SPARC shRNA in a stable manner. Screening of the clones was performed by immunoblot analysis. The 43 kDa band corresponding to SPARC protein was almost completely inhibited in SPARC shRNA-expressing clones in comparison with wild-type and control-transfected clones. The SPARC protein band was only detectable on long exposures in SPARC shRNA-expressing clones (Figure 2A). Two-week additional bands above the SPARC band and an intensive band of about 20 kDa were picked up on the long exposure autoradiographs by the antibody in all lysates (Figure 2A). Their level was not altered. A total of 16 shRNA-expressing clones (8 ASPC, 8 PANC-1) were randomly picked and screened for basal cell growth. Inhibition of endogenous SPARC resulted in enhanced basal cell growth in all but one (P4-20, 101±2% s.e.) clone. The basal growth of ASPC-1 clones A1-6, A1-12, A3-4, A3-13, A4-10, and A4-11 was 130% (±4% s.e.), 119% (±5% s.e.), 137% (±5% s.e.), 130% (±5% s.e.), 139% (±6% s.e.), and 132% (±5% s.e.), respectively, and for PANC-1 clones P3-20, P1-21, P2-9, P3-21, and P4-21 122% (±6% s.e.), 123% (±6% s.e.), 122% (±5% s.e.), 116% (±5% s.e.), and 136% (±2% s.e.), respectively, compared with respective wild-type cells (four separate experiments). The results of control-transfected clones AN-9 and PN-21 as well as of SPARC shRNA-expressing clones A2-7, A3-3, P1-15, and P1-20 are displayed in Figure 2B. Addition of exogenous SPARC (5 *μ*g ml^–1^) to SPARC-deprived cell clones inhibited their basal growth to the same extent as seen in wild-type cells by 29.5% (±5.2% s.e.m.) and 13.9% (±1.1% s.e.m.) in PANC-1 (P1-15) and ASPC-1 (A2-7) clones, respectively.

**Figure 3:**
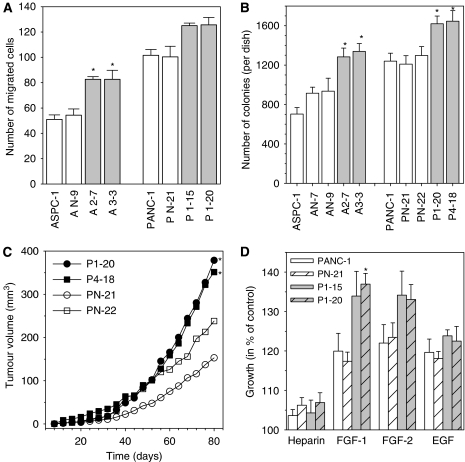
Inhibition of endogenous SPARC and its effect on cell migration, colony-formation, xenograft growth, and FGF-induced proliferation. (**A**) Migration assay. The number of cells that moved to the lower side of the Boyden chamber was determined as described in the legend of Figure 1 for wild-type ASPC-1 and PANC-1 cells, vector alone (control)-transfected clones (AN-9, PN-21), and SPARC shRNA-expressing clones (A2-7, A3-3, P1-15, P1-20), respectively. The results are expressed as mean number (±s.d.) of migrated cells within 36 h of three separate experiments. ^*^P<0.05 compared with wild-type and control-transfected cells. (**B**) Soft-agar assay. Colony-formation of indicated cells was determined after 14 days by staining and counting viable colonies. The results are mean number of colonies per dish (±s.e.) of triplicate determinations from five separate experiments. ^*^P<0.05 compared with wild-type and control-transfected cells. (**C**) Xenograft formation. Indicated PANC-1 cells (10^6^ per site) were subcutaneously injected into nude mice. Tumour growth was followed for 80 days. Eleven sites of 16 injected sites of PN-21 and 6 of 8 of PN-22 control-transfected clones and 12 of 16 of P1-20 and 8 of 8 of P1-15 SPARC shRNA-expressing clones developed tumours. Shown is the mean tumour volume for developed nodules: PN-21 (*n*=11), PN-22 (*n*=6), P1-15 (*n*=8), P1-20 (*n*=12). ^*^P<0.05 compared with PN-21. (**D**) FGF-induced proliferation. Wild-type PANC-1, control-transfected (PN-21), and SPARC shRNA (P1-15, P1-20)-expressing cells were cultured for 48 h in serum-free medium in the presence of heparin alone (1 *μ*g ml^–1^), 5 nM FGF1 or FGF2 in the presence of heparin, or 5 nM EGF. Proliferation was determined by the MTT assay. The results are shown as mean growth (in %) of respective untreated control and are means (±s.e.) of quadruplicate determinations from three separate experiments. ^*^P<0.05 compared with the effects of ligand in wild-type and control-transfected cells.

SPARC shRNA expression enhanced the malignant phenotype of PDAC cells. Inhibition of SPARC resulted in enhanced basal cell migration (Figure 3A), basal colony formation in soft-agar (Figure 3B), and xenograft formation (Figure 3C). Moreover, inhibition of endogenous SPARC expression was accompanied by enhanced proliferative activity of FGF1 and FGF2, but not EGF (Figure 3D).

### Effects of FGFR1 expression on SPARC modulation of FGF1 and FGF2

**Figure 4:**
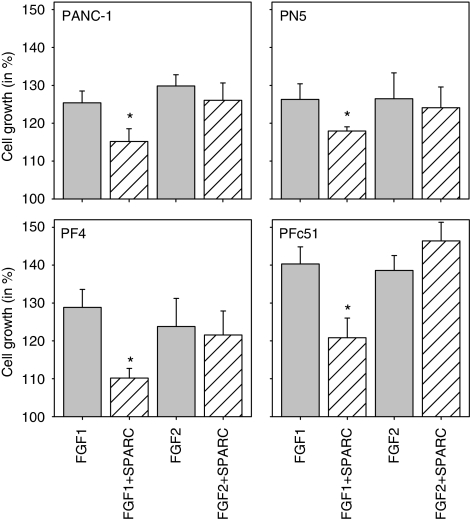
Modulation of FGF1- and 2-induced proliferation by exogenous SPARC in cells over-expressing FGFR1-III isoforms. Wild-type (PANC-1), control-transfected (PN5), FGFR1-IIIb over-expressing (PF4), and FGFR1-IIIc over-expressing (PFc51) cells were incubated for 48 h in serum-free medium with 5 nM FGF1 or FGF2 alone and in the presence of SPARC (5 *μ*g ml^–1^). The results are shown as mean growth (in %) of control (heparin only) and are means (±s.e.) of quadruplicate determinations from three separate experiments. ^*^P<0.05 compared with ligand alone.

Modulation of FGF actions by SPARC was reported to depend on FGFR1 expression (Motamed *et al*, 2003). It is not known, whether there are differences among the existing FGFR1 variants in mediating SPARC-modulated FGF actions. Therefore, we next investigated SPARC modulation of FGF-depended proliferation in regard to FGFR1-III isoform expression. Wild-type PANC-1, control-transfected (PN5), FGFR1-IIIb over-expressing (PF4), and FGFR1-IIIc over-expressing (PFc51) cells were incubated with FGF1 and FGF2 in the presence and absence of SPARC. As reported earlier, over-expression of FGFR1-IIIc (PFc51) resulted in enhanced FGF1- and FGF2-induced proliferation in comparison with wild-type, control-transfected, and FGFR1-IIIb (PF4) over-expressing cells (Figure 4). FGF2 effects were not markedly altered by exogenous SPARC. Irrespective of the FGFR1-III isoform and the degree of FGF1-induced proliferation, the inhibitory effects of SPARC on FGF1-induced proliferation seemed to be slightly more pronounced in FGFR1-III over-expressing clones compared with cells expressing lower levels of FGFR1 (Figure 4). This observation did not reach any significance.

### Effect of FGFR1-III domain expression on SPARC expression

**Figure 5:**
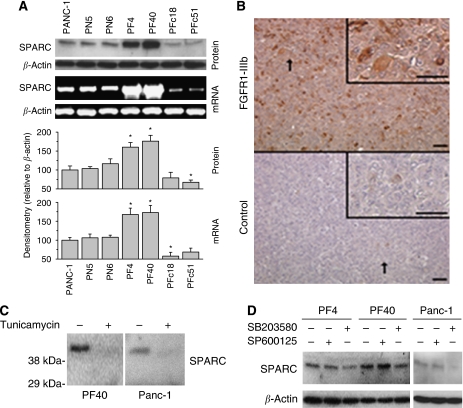
Function of FGFR1-III isoform expression and p38-MAPK activity for SPARC expression. (**A**) SPARC expression in cells over-expressing FGFR1-IIIb and FGFR1-IIIc. The upper panels show representative SPARC immunoblot and PCR analyses in wild-type PANC-1 cells, PN5 and PN6 control-transfected, FGFR1-IIIb over-expressing PF4 and PF40, and FGFR1-IIIc over-expressing PFc18 and PFc51 cells. Densitometry of bands in relation to respective *β*-actin of three separate experiments is shown in the lower panel. The results are mean expression levels (±s.d.) in relation to wild-type PANC-1 (100%) of three separate experiments. ^*^P<0.05 compared with wild-type and control-transfected cells. (**B**) SPARC expression in xenograft tumours. Immunohistochemical analysis of SPARC was performed in FGFR1-IIIb over-expressing (upper panel) and control-transfected (lower panel) tumours (Liu *et al*, 2007b). Representative areas from proliferating areas are shown. The arrow depicts the area of magnification of the inset. Bar=50 *μ*m. (**C**) Glycosylation of SPARC. Incubation of FGFR1-IIIb over-expressing PF4 and wild-type PANC-1 cells with tunicamycin showed that SPARC protein was glycosylated. (**D**) Effects of the p38-MAPK inhibitor SB203580 and the JNK inhibitor SP600125 on endogenous SPARC expression in FGFR1-IIIb over-expressing PF4 and PF40 and wild-type PANC-1 cells. Indicated cells were incubated in the absence (−) and presence (+) of SB203580 (20 *μ*M) or SP600125 (2 *μ*M) for 24 h before analysis.

Over-expression of FGFR1-IIIb inhibited and FGFR1-IIIc enhanced the malignant phenotype of PDAC cells (Liu *et al*, 2007b). In the next step, endogenous SPARC expression in PANC-1 cells over-expressing either FGFR1-IIIb (PF4, PF40) or FGFR1-IIIc (PF18c, PF51c) was characterised. Expression of SPARC was enhanced in FGFR1-IIIb over-expressing clones, whereas SPARC expression was decreased in FGFR1-IIIc over-expressing clones (Figure 5A).

This was confirmed in a xenograft model *in vivo*. Immunohistochemical analysis of SPARC protein expression of tumours over-expressing FGFR1-IIIb (Liu *et al*, 2007b) revealed that SPARC protein was up-regulated in FGFR1-IIIb over-expressing tumours (Figure 5B).

Incubation with tunicamycin, an inhibitor of glycosylation, showed that up-regulated SPARC protein in FGFR1-IIIb over-expressing PANC-1 cells was a glycosylated protein similar to wild-type cells (Figure 5C).

The mechanism of SPARC up-regulation in FGFR1-IIIb-expressing clones is unclear. We showed recently that FGFR1-IIIb over-expression was accompanied by up-regulation of p38-MAPK and JNK activities (Liu *et al*, 2007a, 2007b). Incubating FGFR1-IIIb over-expressing PF4 and PF40 cells with increasing concentrations of the p38-MAPK inhibitor SB203580 (10, 20, and 40 *μ*M) and the JNK inhibitor SP600125 (1, 2, and 4 *μ*M) revealed that SB203580 reduced expression of SPARC, whereas SP600125 was without effect. In Figure 5D, the effects of 20 *μ*M SB203580 and of 2 *μ*M SP600125 are shown for FGFR1-IIIb over-expressing clones and wild-type PANC-1 cells. Densitometric analysis in relation to *β*-actin of three separate experiments revealed that SB203580 reduced SPARC expression in wild-type PANC-1, PF4, and PF40 cells by 65% (±8% s.d.), 47% (±5% s.d.), and 62% (±9% s.d.), respectively. SP600125 was without significant effect.

### Interactions of PDAC cells and stromal cells

**Figure 6:**
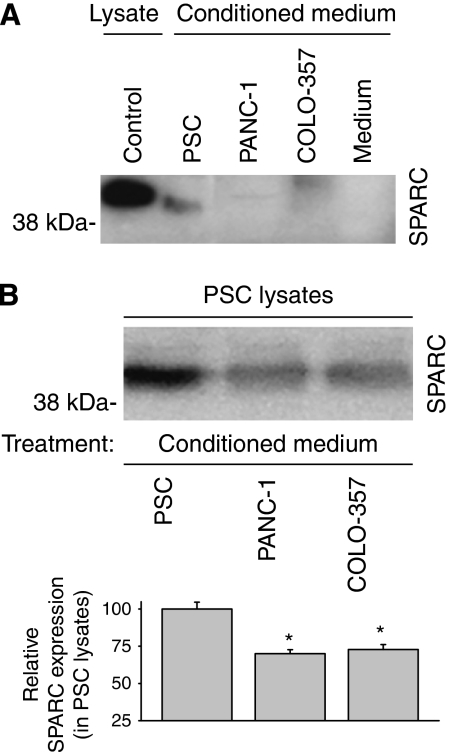
Secretion and regulation of SPARC protein expression in human pancreatic stellate cells (PSCs). (**A**) Detection of SPARC protein in conditioned medium. Conditioned medium of PSCs, PANC-1, and COLO-357 was collected for 48 h and subjected to immunoblot analysis after pull down of heparin-binding proteins by heparin sepharose. Cell lysates of PSCs were used as positive control (control), medium alone as negative control (medium). (**B**) Regulation of endogenous SPARC expression in PSCs. PSCs were incubated (treatment) with conditioned medium of PSCs, COLO-357, or PANC-1 cells for 48 h. SPARC immunoblot analysis of total PSC cell lysates after incubation is shown in the upper panel, relative SPARC protein expression after densitometric analysis in the lower panel. The results in the lower panel are mean SPARC levels (±s.d.) in relation to PSC (100%) of three separate experiments. ^*^P<0.05 compared with control (PSC).

Human PSCs expressed high levels of SPARC (Figure 1A). SPARC was also detectable in the pull down of heparin-binding proteins of conditioned medium of PSCs, but not of COLO-357 and PANC-1 pancreatic cancer cells (Figure 6A). To investigate whether SPARC expression can be altered by paracrine mechanisms, PSCs were incubated with conditioned medium harvested from PSCs and pancreatic cancer cells (Figure 6B). Endogenous SPARC expression in PSCs was reduced by conditioned medium of COLO-357 and PANC-1 cells added to PSCs for 48 h, but not by conditioned medium of PSCs (Figure 6B). Conditioned medium of PSCs did not alter SPARC expression in PANC-1 and ASPC-1 cells (data not shown).

## Discussion

SPARC is a matricellular protein with antiproliferative and counteradhesive functions (Yan and Sage, 1999). Its function in cancer is discussed in a very controversial manner (Clark and Sage, 2008; Podhajcer *et al*, 2008; Tai and Tang, 2008). In certain types of cancers, such as melanomas and gliomas, SPARC is associated with a highly aggressive tumour phenotype. In others, mainly ovarian, neuroblastomas, and colorectal cancers, SPARC may function as a tumour suppressor (Tai and Tang, 2008). We showed in this study that exogenous SPARC can inhibit cell proliferation, single cell movement, and migration of cultured PDAC cells. These exogenous SPARC functions were independent of endogenous SPARC expression. As reported (Sato *et al*, 2003; Guweidhi *et al*, 2005), the majority of the cell lines did not express endogenous SPARC, probably as a result of aberrant hypermethylation (Sato *et al*, 2003; Cheetham *et al*, 2008).

Inhibition of endogenous SPARC in cultured human PDAC cells by small hairpin RNA enhanced cell proliferation, migration, colony formation, and xenograft formation. These results indicate that endogenous SPARC may act as a tumour suppressor in PDAC cells, a function SPARC also has in ovarian, neuroblastomas, and colorectal cancers (Tai and Tang, 2008). This function of a tumour suppressor is also supported by our finding that inhibition of endogenous SPARC resulted in a doubling of the mitogenic activity of FGF1 and FGF2, two ligands of the FGF family known to be over-expressed in the majority of pancreatic cancers and to correlate with poor prognosis (Yamanaka *et al*, 1993).

The presence of FGFR1 is important for mediating SPARC functions in endothelial cells and skeletal myoblasts (Motamed *et al*, 2003). All cell lines used in our study expressed various levels of FGFR1 (Kornmann *et al*, 2002). Inhibition of FGF1-induced proliferation by exogenous SPARC was enhanced in FGFR1-III over-expressing cells compared with controls. This effect was independent of the FGFR1-III isoform. Thus, exogenous SPARC actions depend on the FGFR1 levels, but are independent of the domain III isoform expression.

Down-regulation of SPARC in pancreatic cancer cells is believed to depend on DNA methylation (Sato *et al*, 2003; Guweidhi *et al*, 2005; Infante *et al*, 2007; Cheetham *et al*, 2008). Another reason contributing to the down-regulation of SPARC in human pancreatic cancer cells may be the over-expression of FGFR1-IIIc. FGFR1-IIIc is the predominant FGFR1 isoform expressed in PDAC (Kornmann *et al*, 2002). FGFR1-IIIb usually expressed in cells of epithelial origin is almost absent in PDAC (Kornmann *et al*, 2002). Re- or over-expression of FGFR1-IIIb in PDAC cells resulted in a marked up-regulation of endogenous SPARC *in vitro* and *in vivo*. In contrast, an additional over-expression of FGFR1-IIIc further lowered endogenous SPARC expression. We recently showed that over-expression of FGFR1-IIIb in PDAC cells reverted the malignant phenotype inhibiting proliferation, single cell movement, and migration *in vitro*, as well as xenograft formation and growth *in vivo* (Liu *et al*, 2007b). On the other hand, it is well known that over-expression of FGFR1-IIIc enhances the malignant phenotype of PDAC (Wagner *et al*, 1998; Kornmann *et al*, 2002).

FGFR1-IIIb over-expression in PDAC cells resulted in strong p38-MAPK and JNK activitation (Liu *et al*, 2007a, 2007b). In this study, we also investigated the effects of specific inhibitors of these kinases on endogenous SPARC levels. Our results showed that endogenous SPARC levels could be down-regulated by inhibition of p38-MAPK, but not of JNK. Therefore, our observations suggest that modulation of endogenous SPARC expression may be one of the mechanisms resulting in the different phenotypes seen for the FGFR1-III domain variants and that the observed FGFR1-IIIb-induced induction of endogenous SPARC is mediated through p38-MAPK.

Recent studies investigating SPARC expression in human pancreatic tissues reported high levels of SPARC in the surrounding stromal tissue harbouring fibroblasts and PSCs, whereas SPARC was often absent in the cancer cells (Guweidhi *et al*, 2005; Infante *et al*, 2007). High SPARC expression in the stroma portended a poor patient prognosis (Infante *et al*, 2007). We showed in our study that PSCs express higher levels of endogenous SPARC than cultured PDAC cells and that SPARC is detectable in the conditioned medium of PSCs. Our study also revealed that conditioned medium of pancreatic cancer cells down-regulated endogenous SPARC expression of PSCs. In contrast, co-culture of fibroblasts in the presence of PDAC cells augmented SPARC expression in fibroblasts (Sato *et al*, 2003), suggesting that high SPARC expression in the tumour stroma may be mainly a result of augmented SPARC expression in stromal fibroblasts.

In summary, we showed that inhibition of endogenous SPARC enhances the malignant phenotype of PDAC cells and showed that endogenous SPARC expression is regulated by FGFR1 domain III isoform expression. On the basis of these observations, we conclude that endogenous SPARC levels can contribute to the reversion of the malignant phenotype and may, therefore, act as a tumour suppressor in human PDAC cells. Future studies in human pancreatic cancer could aim at the design of treatment strategies specifically targeting SPARC–FGFR1 interactions.
